# Exoproteomic profiling uncovers critical determinants for virulence of livestock-associated and human-originated *Staphylococcus aureus* ST398 strains

**DOI:** 10.1080/21505594.2020.1793525

**Published:** 2020-07-29

**Authors:** Xin Zhao, Monika A. Chlebowicz-Flissikowska, Min Wang, Elias Vera Murguia, Anne de Jong, Dörte Becher, Sandra Maaß, Girbe Buist, Jan Maarten van Dijl

**Affiliations:** a Department of Medical Microbiology, University of Groningen, University Medical Center Groningen, Groningen, The Netherlands; b Department of Molecular Genetics, University of Groningen, Groningen Biomolecular Sciences and Biotechnology Institute, Groningen, The Netherlands; c Institute of Microbiology, University of Greifswald, Greifswald, Germany

**Keywords:** *S. aureus*, ST398, exoproteome, virulence, *Galleria mellonella*, Hela cells

## Abstract

Staphylococcus aureus: with the sequence type (ST) 398 was previously associated with livestock carriage. However, in recent years livestock-independent *S. aureus* ST398 has emerged, representing a potential health risk for humans especially in nosocomial settings. Judged by whole-genome sequencing analyses, the livestock- and human originated strains belong to two different *S. aureus* ST398 clades but, to date, it was not known to what extent these clades differ in terms of actual virulence. Therefore, the objective of this study was to profile the exoproteomes of 30 representative *S. aureus* ST398 strains by mass spectrometry, to assess clade-specific differences in virulence factor secretion, and to correlate the identified proteins and their relative abundance to the strains’ actual virulence. Although the human-originated strains are more heterogeneous at the genome level, our observations show that they are more homogeneous in terms of virulence factor production than the livestock-associated strains. To assess differences in virulence, infection models based on larvae of the wax moth *Galleria mellonella* and the human HeLa cell line were applied. Correlation of the exoproteome data to larval killing and toxicity toward HeLa cells uncovered critical roles of the staphylococcal Sbi, SpA, SCIN and CHIPS proteins in virulence. These findings were validated by showing that *sbi* or *spa* mutant bacteria are attenuated in *G. mellonella* and that the purified SCIN and CHIPS proteins are toxic for HeLa cells. Altogether, we show that exoproteome profiling allows the identification of critical determinants for virulence of livestock-associated and human-originated *S. aureus* ST398 strains.

## Introduction

*Staphylococcus aureus* is one of today’s major nosocomial and community-acquired pathogens. Infections caused by this pathogen are associated with substantial morbidity and mortality, and *S. aureus* therefore represents a major threat for public health [1]. Nevertheless, about 20–30% of the healthy human population carries *S. aureus* asymptomatically [1]. Infections with *S. aureus* have become increasingly difficult to treat due to the emergence of antibiotic resistant lineages, as underpinned by methicillin-resistant *S. aureus* (MRSA) [2].

Over the past 20 years, the epidemiology of MRSA has changed dramatically. Initially, MRSA was almost exclusively identified as a hospital-acquired (HA) pathogen. Subsequently, community-associated (CA) MRSA lineages were identified that caused severe infections in individuals with no apparent healthcare contacts [3]. Over the last decade, livestock-associated (LA) MRSA was identified in livestock and individuals exposed to livestock, especially in pig farms [4]. *S. aureus* with the sequence type 398 (ST398), which belongs to the clonal cluster 398 (CC398), is the most prevalent livestock-associated lineage causing zoonotic disease in Europe, North America, and Asia [5].

Intriguingly, recent whole-genome sequence analyses have revealed that the current *S. aureus* CC398 isolates represent two distinct phylogenetic clades, where one harbors truly livestock-associated strains (here designated as LA-ST398), while the other represents strains originating from humans (here referred to as human-originated ST398) [6–8]. The LA-ST398 strains can be exchanged between livestock and humans, where they may cause moderate infections and occasionally severe infections [9,10]. In contrast, the human-originated ST398 strains are able to spread by human-to-human transmission, and have been implicated in acute and even fatal infections [8,11]. Some distinguishing features of the two CC398 clades have been identified [6–8]. In particular, most LA-ST398 strains are MRSA and tetracycline-resistant, whereas the human-originated ST398 isolates are mostly methicillin-sensitive *S. aureus* (MSSA) and tetracycline-sensitive [6,8,11]. Further, human-originated isolates often harbor the β-hemolysin (*hlb*) converting prophage ϕSa3, which encodes the immune evasion cluster (IEC) genes *chp* and *scn*. The respective encoded proteins, the neutrophil chemotaxis-inhibiting protein (CHIPS) and the staphylococcal complement inhibitor (SCIN), allow *S. aureus* to evade phagocytosis and killing by human neutrophils [12]. In addition, the ϕSa3-borne IEC gene *sak*, encoding the defensin inhibitor staphylokinase, is present in some subclades of the human-originated CC398 [6]. The phage-mediated acquisition of the IEC genes may thus explain how some CC398 strains adapt to the human host, and why these strains can cause more severe infections. Lastly, a recent study showed that the LA-ST398 and human-originated CC398 isolates possess distinctive single-nucleotide polymorphisms (canSNP_748, canSNP_1002 and canSNP_3737) [7].

To date, the majority of studies on *S. aureus* ST398 isolates were focused on variations in the core genome and mobile genetic elements (MGEs). In contrast, variations in the actual production of virulence factors have not been investigated systematically. This raises the question how similar or different isolates belonging to the LA-ST398 or human-originated ST398 clades are in terms of virulence factor production? An answer to this question would be relevant for assessment of the infection risks associated with particular *S. aureus* ST398 isolates. High-throughput proteomics is a particularly powerful tool to explore bacterial virulence factor production, especially since most virulence factors are secreted into the extracellular milieu of the bacteria [13]. Upon their export from the bacterial cytoplasm, these virulence factors play crucial roles in the different stages of host infection, especially adhesion, colonization, immune evasion and invasion [14–16]. Accordingly, the bacterial exoproteome should be regarded as a major reservoir for virulence factors [15,17].

The aim of the present study was to investigate variations in the exoproteomes of different LA-ST398 and human-originated ST398 isolates, and to assess possible correlations between identified exoproteome differences and virulence. To this end, we profiled the exoproteomes of 30 different *S. aureus* ST398 isolates, which are representative for the two major clades that make up the CC398. Further, possible implications of the observed exoproteome variations for overall virulence were evaluated by infection experiments with larvae of the greater wax moth *Galleria mellonella*, which represent a facile model to assess bacterial virulence [18–21]. In addition, the cytotoxic effects of the different *S. aureus* ST398 isolates were investigated using a human HeLa cell infection model. In brief, our results show that the investigated LA-ST398 isolates display a higher level of exoproteome heterogeneity than the human-originated ST398 isolates. This finding is mirrored by a more homogeneous behavior of the human-originated isolates in the two infection models, where the human-originated isolates display on average higher virulence and cytotoxicity. While it remains difficult to directly associate particular patterns of produced virulence factors with the actual virulence of particular ST398 isolates observed in our two infection models, clear associations between the Sbi and SpA proteins and larval killing, and between the CHIPS and SCIN proteins and cytotoxicity in HeLa cells could be demonstrated.

## Materials and methods

### Bacterial isolates

Relevant features of the 30 *S. aureus* strains with ST398 used in this study are presented in Table 1. All strains were isolated, typed and whole-genome sequenced by Illumina sequencing as previously described [22], allowing their phylogenetic distinction as LA-ST398 or human-originated ST398 using RAxML v7.0.4.

**Table 1.** Phylogeny and main characteristics of the 30 investigated *S. aureus* ST398 study isolates. The phylogeny was based on the core genomes of the different *S. aureus* ST398 isolates included in the present study. The presence (+) or absence (-) of particular virulence genes is indicated. Col, colonization; inf, infection; ND, not determined.

	

### Bacterial cultivation and collection of extracellular proteins

*S. aureus* strains were grown overnight at 37°C in tryptic soy broth (TSB; OXOID, Basingstoke, UK) under vigorous shaking (115 rpm) in a water bath. The cultures were then diluted into 10 mL pre-warmed Roswell Park Memorial Institute 1640 (RPMI) medium supplemented with 2 mM glutamine (GE Healthcare/PAA, Little Chalfont, United Kingdom) to an optical density at 600 nm (OD_600_) of 0.1 and cultivation was continued under the same conditions. Exponentially growing cells with an OD_600_ of ±0.5 were again diluted into 15 mL of fresh pre-warmed RPMI 1640 medium to a final OD_600_ of 0.1 and their cultivation was continued until an OD_600_ of ±1.2 was reached, which corresponds to the stationary growth phase. Then, cells were separated from the growth medium by centrifugation, and proteins in the growth medium were precipitated overnight at 4°C using 10% trichloroacetic acid (Sigma-Aldrich, St. Louis, USA). The precipitated proteins were collected by centrifugation, washed once with ice-cold acetone, dried at room temperature, and stored at −20°C until further use.

### Sample preparation for the extracellular proteome analysis by LC-MS/MS

The collected extracellular proteins were processed for mass spectrometry (MS) analysis essentially as described previously [23]. In brief, the dried protein pellets were re-suspended in 50 mM ammonium bicarbonate buffer (Fluka, Buches, Switzerland) and reduced with 500 mM dithiothreitol (Duchefa Biochemie, the Netherlands) for 45 min at 60°C. The samples were then alkylated with 500 mM iodoacetamide (Sigma-Aldrich) and incubated for 15 min in the dark at room temperature. 100 ng of sequencing grade modified trypsin (Promega, Madison, USA) were added and the mixture was incubated overnight at 37°C under continuous shaking at 250 rpm to completely digest the proteins. Subsequently, the tryptic peptides were acidified with a final concentration of 0.1% trifluoroacetic acid (Sigma-Aldrich, St. Louis, USA) for 45 min at 37°C to inactivate the trypsin. The digested peptides were purified using ZipTips-C18 material (Millipore, Billerica, USA) and eluted in 60% acetonitrile/0.1% MS-acetic acid. Lastly, the eluted peptides were dried in a SpeedVac (Eppendorf, Hamburg, Germany) at room temperature and stored at 4°C until further use.

### Mass spectrometry and data analyses

Purified peptides were identified by reversed-phase liquid chromatography coupled to electrospray ionization MS using an LTQ Orbitrap XL (Thermo Fisher Scientific, Waltham, MA) as described by Stobernack *et al*. [24]. Sorcerer-SEQUEST 4 (Sage-N Research, Milpitas, USA) was applied for database searching, and raw data files were searched with Sequest against a target-decoy database with a set of common laboratory contaminations. The database used for protein identifications was based on whole genome sequences of the 30 *S. aureus* study isolates. The RAST annotation file of the 30 study isolates was used to create a non-redundant database comprising protein sequences of all isolates. This database includes a total of 3915 protein sequences with connected gene names and Uniprot identifiers. Protein sequences that differed in at least 1 amino acid were included in this database. Finally, the gene names and uniprot identifiers were added. Validation of MS/MS-based peptide and protein identification was performed with Scaffold V4.7.5 (Proteome Software, Portland, USA), and peptide identifications were accepted if they exceeded the specific database search engine thresholds. SEQUEST identifications required at least deltaCn scores of greater than 0.1 and XCorr scores of greater than 2.2, 3.3 and 3.75 for doubly, triply and all higher charged peptides, respectively. Protein identifications were accepted if at least 2 identified peptides were detected with the above-mentioned filter criteria in 2 out of 3 biological replicates. With these filter parameters, no false-positive hits were obtained, as was verified by a search against a concatenated target-pseudo-reversed decoy database. Quantitative values of protein abundances were obtained by summing up all spectra associated with a specific protein within a sample, which includes also those spectra that are shared with other proteins. To allow comparisons, spectral counts were normalized by applying a scaling factor for each sample to each protein adjusting the values to normalized spectral counts [25]. Of note, some proteins are easier to detect than others, which may affect the comparison of abundance levels of different proteins. The normalized spectral count data were exported from Scaffold and to Microsoft Excel for further analysis (Supplementary Table S1).

### Assessment of virulence with a *Galleria mellonella* infection model

*G. mellonella* larvae in their final instar stage were purchased (Frits Kuiper, Groningen, Netherlands), maintained on wood chips in the dark and used within 7 days of receipt. Larvae of ~250 mg in weight and 2 cm in length were employed in all assays. Ten randomly chosen larvae were used for assessing the virulence of each investigated *S. aureus* ST398 isolate, with each infection experiment repeated at least 3 times. Before inoculation into *G. mellonella, S. aureus* cells were harvested in the exponential growth phase from a culture in RPMI, following the same protocol for bacterial culturing as was used for the proteome analyses. An insulin pen (HumaPen LUXURA® HD, Indianapolis, USA) [26] was used to inject 10 μl aliquots of a diluted bacterial suspension (2.5 × 10^5^ CFU) into the hemocoel via the last proleg. Control larvae were either injected with 10 μl of PBS in order to monitor the impact of physical trauma, or underwent no manipulation whatsoever. After injection, the larvae were kept in petri dishes in the dark at 37°C, and mortality was monitored after 48 h post infection. Larvae were considered dead when they displayed no movement in response to touch.

### Assessment of staphylococcal cytotoxicity with a HeLa cell infection model

Human cervical cancer HeLa cells were cultured in DMEM-GlutaMAX^TM^ medium (Gibco, UK) supplemented with 10% fetal calf serum (Sigma-Aldrich, USA) using a humidified CO_2_ incubator (37°C, 5% CO_2_). For infection experiments, aliquots of 3 × 10^4^ HeLa cells were resuspended in 100 μL fresh DMEM-GlutaMAX^TM^ medium in wells of a 96-well tissue culture plate and the plate was subsequently incubated for 24 h (37°C, 5% CO_2_). The cells were then infected at a multiplicity of infection (MOI) of 50 with PBS-diluted *S. aureus* cells that had been cultured in RPMI medium as described above. Next, the infected cells were incubated for 3 h (37°C, 5% CO_2_). Non-internalized bacteria were eliminated 3 h post-infection by two washes with PBS and the subsequent addition of 20 μg/ml of lysostaphin (AMBI Products, NY, USA). To quantify the viability of the infected HeLa cells, the 3-(4,5-Dimethylthiazol-2-yl)-2,5-Diphenyltetrazolium Bromide (MTT; Sigma Aldrich, NL) dye reduction assay was applied. To this end, the infected HeLa cells were incubated with MTT at a final concentration of 0.5 mg/ml for 3 h (37°C, 5% CO_2_). Lastly, the cells were resuspended in 150 μL of acidic isopropanol to solubilize crystals of formazan produced by mitochondrial activity, and the absorbance at 570 nm was determined using a BioTek Synergy 2 plate reader. In this MTT assay, the amount of color produced is proportional to the number of viable cells. The cytotoxicity of individual *S. aureus* isolates was expressed as the absorbance at 570 nm relative to the control, where HeLa cells were incubated in the absence of infecting *S. aureus* cells.

### Expression of staphylococcal SCIN, CHIPS and LysM proteins in *L. lactis*

To investigate the cytotoxicity of the SCIN and CHIPS proteins of *S. aureus*, these proteins were expressed with a His_6_-tag in *Lactococcus lactis* as described before [27]. As a control, the LysM domain of the *S. aureus* protein Sle1 was also expressed with a His_6_-tag in *L. lactis* and purified. To this end, the LysM domain-encoding sequence was amplified from chromosomal DNA of *S. aureus* NCTC8325 (isolated with the innuPREP kit; Analytik Jena), using the primers LysM-F (5ʹ- ATATGGATCCGCTACAACTCACACAGTAAAAC) and LysM-R (5ʹ- ATATGCGGCC GCTTAGTTCGTA GATGCATTACCAG) and the PWO-polymerase (Roche Diagnostics). The PCR-amplified LysM domain-encoding fragment was cleaved with *Not*I and *BamH*I (New England Biolabs), and ligated to plasmid pNG4210 that was cleaved with the same restriction endonucleases. The resulting ligation mixture was used to transform electrocompetent *L. lactis* PA1001 [28]. *L. lactis* containing pNG4210-*lysM* were selected on M17 plates supplemented with 0.5% glucose (w/v), 0.5 M sucrose and chloramphenicol (5 μg/ml) at 30°C and the correct plasmid construction was verified by sequencing (Eurofins MWG Operon, Ebersberg, Germany).

To produce the His_6_-tagged SCIN, CHIPS and LysM protein, *L. lactis* cells carrying the respective expression plasmids were grown in M17 broth (Oxoid Limited, Hampshire, UK) supplemented with 0.5% glucose (w/v) and chloramphenicol (5 μg/ml). The production of SCIN, CHIPS or LysM in exponentially growing cultures of *L. lactis* (OD_600_ ≈ 0.5) was induced by the addition of nisin (3 ng/ml, SigmaAldrich, St. Luis, MO). Growth medium fractions were harvested after overnight culturing at 30°C, and the His_6_-tagged proteins were purified from the supernatant fractions using HisLink™ Protein Purification Resin (Promega Corporation, Madison, WI. USA). Lastly, the three purified proteins were analyzed by LDS-PAGE and Simply Blue Safe Staining.

### Bioinformatic and statistical analyses

Virulence genes were identified from the genome sequences of the 30 investigated ST398 strains using the ABRicate program with VFDB database (https://github.com/tseemann/abricate). Bioinformatic tools including TMHMM (version 2.0) [29], SignalP (version 4.1) [30], PsortB (version 3.0.2) [31], and SecretomeP (version 2.0) [32] were used for prediction of the subcellular location of proteins identified by MS analyses. Gene annotations and functional categories were assigned using TIGRfam (version 15.0) and the AureoWiki database (http://aureowiki.med.uni-greifswald.de). For visualization of identified protein functions and the respective protein abundances, Voronoi treemaps were built using Paver version 2.1 (Decodon GmnH, Greifswald, Germany) as described [33]. To assess the overall relationships between isolates of two ST398 subgroups in terms of their exoproteome profiles, a principal component analysis (PCA) was performed based on the MS data using ClustVis [34]. Significant differences in protein spectral counts between isolates belonging to sub-clades were assessed by multiple t tests and a subsequent Holm-Sidak correction to adjust the P-values. The statistical significance of differences in the killing of *G. mellonella* larvae by the *S. aureus* ST398 was assessed by Wilcoxon tests. The statistical significance of the observed differences in the killing of HeLa cells by was assessed using one-way ANOVA with Dunnett’s multiple-comparison test.

### Biological and chemical safety

*S. aureus* is a biosafety level 2 microbiological agent and was accordingly handled following appropriate safety procedures. All experiments involving live *S. aureus* bacteria and chemical manipulations of *S. aureus* protein extracts were performed under appropriate containment conditions, and protective gloves were worn. All chemicals and reagents used in this study were handled according to the local guidelines for safe usage and protection of the environment.

### Data availability

The mass spectrometry data are deposited in the ProteomeXchange repository PRIDE (https://www.ebi.ac.uk/pride/). The dataset identifier is PXD013951.

## Results

### Features of the selected *S. aureus* ST398 study isolates

The 30 *S. aureus* isolates used in this study were selected from a previous collection of 182 ST398 isolates that had been derived from food, pigs, pig handlers or humans (Table 1). The isolates in this collection had been characterized by multi-locus sequence typing and *spa*-typing. In addition, the whole genome sequences of these isolates had been determined. This allowed the distinction of LA-ST398 and human-originated ST398 isolates in the collection, which were phylogenetically divided into two major clades and several sub-clades as shown in the rooted phylogenetic tree in Table 1. In the selection of representative isolates from the different clades and sub-clades of *S. aureus* ST398 for our present proteome analyses, we strived to cover the majority of the identified sub-clades, as well as isolates showing the largest diversity within the different sub-clades. Further, we limited the number of selected study isolates to a total of 30 isolates for reasons of technical feasibility. Specifically, seventeen of the selected strains are LA-ST398 strains isolated in Europe, Canada or the USA from pigs or pig handlers. The other thirteen strains belong to the human-originated ST398 clade. Eleven of the latter strains had been collected from humans without livestock contact, and two had been isolated from food and pig. Most human-originated ST398 strains were isolated in China (Table 1). Further, most of the selected LA-ST398 strains were MRSA, while most human-originated ST398 strains were MSSA. Only three of the human-originated ST398 strains were MRSA, of which two had been isolated in the Netherlands. Lastly, all of the LA-ST398 strains harbor the *hlb* gene, while the majority of the human-originated ST398 strains carry the IEC genes *chp, scn* and *sak*. Only three strains belonging to the human-originated sub-clade 1.6b carried the *pvl* genes (Table 1), while the *tsst-1* gene for the toxic shock syndrome toxin was absent from the selected strains. Altogether, the selected strains represent the diversity as encountered in the global *S. aureus* ST398 population.

### LA-ST398 strains display higher exoproteome heterogeneity than human-originated ST398 strains

**Figure 1.**
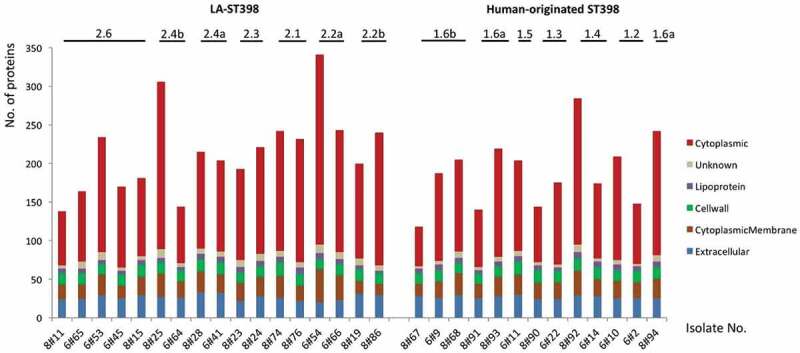
Numbers of identified extracellular proteins of the investigated *S. aureus* ST398 isolates and their predicted subcellular locations. For all identified extracellular proteins of the investigated strains, the subcellular locations were predicted bioinformatically. Subsequently, the respective numbers of proteins assigned to the different subcellular locations were determined per strain (marked in color code). The different clades of the investigated LA-ST398 and human-originated *S. aureus* ST398 strains are indicated.

To identify possible exoproteome variations across the 30 selected LA-ST398 and human-originated ST398 strains, they were cultured in RPMI medium since a previous study had shown that the global gene expression profiles of *S. aureus* grown in RPMI medium or in human plasma are highly similar [35]. Further, secreted proteins of the selected strains were collected in the early stationary growth phase, where the majority of virulence factors is produced and secreted [36]. To this end, the growth medium was separated from the bacterial cells by centrifugation and the secreted proteins in the growth medium fractions were precipitated with trichloroacetic acid. The thus collected extracellular proteins were then analyzed by LC-MS/MS analysis and label-free quantification. In total, 495 different proteins were identified in the combined exoproteome samples of all 30 investigated strains. Among the 495 extracellular proteins, 40 proteins were found to be produced by all 30 strains and a further 80 proteins were identified in at least 80% of these strains. As judged by label-free quantification, these 120 most common proteins contribute to 73.5% of the identified protein abundance in the combined exoproteomes of the 30 investigated strains, high-lighting their dominant expression in the ST398 lineage of *S. aureus* (Supplementary Table S2, Sheet 1). Importantly, these highly conserved and abundant proteins thus represent the core exoproteome of the two main ST398 clades. In contrast, the remaining 375 proteins were highly variable, representing only 26.5% of the total exoproteome abundance. Lastly, 131 of the latter proteins were uniquely identified in only one or two strains. All identified proteins as assigned to the core or variable exoproteomes are listed in Supplementary Table S2 (Sheet 2), and the total numbers of identified protein per strain are indicated in Figure 1.

With the help of different bioinformatic tools, the subcellular location of the identified proteins was predicted. The vast majority of the identified extracellular proteins (n = 313) were predicted as cytoplasmic proteins (63%). This is a commonly observed feature among staphylococci and the respective proteins are now mostly referred to as extracellular cytoplasmic proteins (ECPs) [37]. Furthermore, 52 proteins (11%) were predicted to have an extracellular localization based on the presence of a secretory signal peptide, 64 (13%) were predicted membrane proteins, 21 (4%) were predicted to be cell wall-associated, and 12 proteins (2%) were predicted lipoproteins. Interestingly, the predicted subcellular location of the different proteins suggests that the proportion of cell wall proteins (16%) is higher in the core exoproteome than in the variable exoproteome (2%). Conversely, the variable exoproteome includes a higher proportion of cytoplasmic proteins (66%) than the core exoproteome (55%) (Supplementary Table S2, Sheet 2).

The numbers of identified proteins and their predicted subcellular location for each of the 30 investigated ST398 strains are indicted separately in Figure 1. The results show that the numbers of identified proteins differed largely between the strains belonging to the two *S. aureus* ST398 clades. This was mostly due to large variations in the numbers of identified ECPs. Particularly, the numbers of ECPs in the investigated LA-ST398 strains ranged from 70 (8#11) to 246 (6#54), and for the human-originated ST398 strains from 51 (8#67) to 189 (8#92). Notably, on average, the numbers of detected ECPs were higher in the LA-ST398 isolates (n = 136) than in the human-originated ST398 strains (n = 110). Conversely, no major differences between the LA-ST398 and human-originated ST398 strains were observed in the numbers of predicted “genuine” extracellular proteins that possess signal peptides for export from the cytoplasm. Altogether, these data imply that the investigated LA-ST398 and human-originated ST398 strains show a somewhat different behavior in terms of the numbers of detectable ECPs.

**Figure 2.**
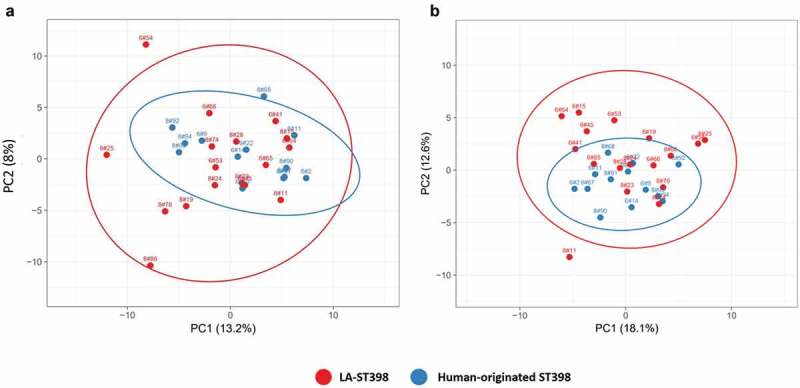
Principal component analysis (PCA) based on the normalized spectral counts of identified extracellular proteins. Two-dimensional PCA plots are displayed for extracellular proteins of the investigated LA-ST398 and human-originated *S. aureus* strains. The PCA analysis was performed on (a) all identified extracellular proteins, and (b) all identified extracellular proteins except the extracellular cytoplasmic proteins (ECPs).

Using the identified extracellular proteins from the LC-MS/MS analysis, as well as the respective label-free quantification data, a principal component analysis (PCA) was performed to elucidate the overall exoproteome relationships among isolates from the two distinct phylogenetic ST398 subgroups. Of note, this PCA analysis was based on the normalized spectral counts of the 407 proteins that were identified both in the LA-ST398 and the human-originated groups of strains. As shown in Figure 2(a), this revealed that the investigated LA-ST398 strains are relatively more heterogeneous with respect to their exoproteome abundance signatures than the human-originated ST398 strains. This observation was unexpected since the phylogeny of the investigated ST398 strains, as based on core genome comparisons, implies a higher diversity amongst the human-originated ST398 strains (Table 1). The latter is all the more remarkable as many of the LA-ST398 strain were isolated from very diverse reservoirs in different geographical regions.

To investigate whether the ECPs impact on the observed exoproteome relationships, a PCA was also performed on all proteins shared between the LA-ST398 and human-originated ST398 groups of strains, but without the ECPs. Interestingly, this PCA based on 182 common proteins revealed an even higher exoproteome variance for the LA-ST398 isolates (Figure 2(b)). Moreover, it can be concluded that the main distinction between the two ST398 subgroups is based on differences in the exoproteome abundance of typical extracytoplasmic proteins that are exported from the cytoplasm with the aid of a signal peptide. These include membrane proteins, lipoproteins, cell wall-bound proteins and proteins that are secreted into the extracellular milieu.

### The exoproteomes of LA-ST398 and human-originated ST398 strains serve distinct roles in metabolism and pathogenesis

**Figure 3.**
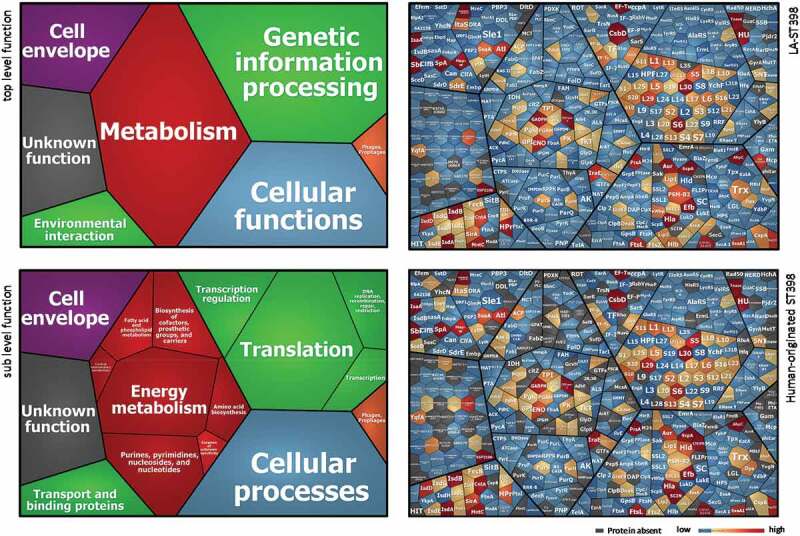
Functional categories and relative abundance of identified extracellular proteins from the investigated LA-ST398 and human-originated ST398 *S. aureus* strain. Voronoi treemaps in the panels on the left show the “top level functions” (TIGRfam level 1) and “sub level functions” (TIGRfam level 2). The different functional categories are marked in different colors, and the size of each functional category is proportional to the number of identified proteins with the respective function. Voronoi treemaps on the right represent the relative abundance of individual extracellular proteins from LA-ST398 strains (top-right panel) or human-originated ST398 strains (bottom-right panel). Each protein is represented by a polygon-shaped tile and its relative average protein abundance is indicated in color code.

To determine the overall exoproteome functions of the investigated *S. aureus* ST398 strains, a functional classification was performed based on the respective TIGRfam and Aureowiki annotations. As shown by the Voronoi treemaps in Figure 3 (panels on the left), the 495 identified extracellular proteins can be grouped according to seven top-level functions and sixteen sub-level functions. The vast majority of identified extracellular proteins were involved in metabolism (27.4%), followed by genetic information processing (26.8%), cellular functions (19.8%), cell envelope (9.3%) and environmental interaction (5.2%). Figure 3 includes also Voronoi treemaps that display the relative abundances of different identified proteins corresponding to the different functional categories for the LA-ST398 strains (upper panel on the right) and human-originated strains (lower panel on the right). The most abundant proteins in both groups of strains are the well-characterized immunodominant staphylococcal antigen A (IsaA), the DNA-binding protein HU, the immunoglobulin-binding protein (Sbi), the extracellular fibrinogen-binding protein (Efb), thermonuclease, and the iron-regulated surface determinant proteins A and B (IsdA, IsdB). These proteins all belong to the ST398 core exoproteome, displaying a diverse range of functions, such as iron acquisition and metabolism, cell wall and capsule biogenesis, DNA metabolism, and immune evasion. Their dominant expression as observed in this study is likely to be important for most *S. aureus* isolates as has been described before [38–40]. Among the 52 identified typical secretory proteins, 27 are known toxins and superantigens, while 6 are involved in pathogenicity and host colonization. Notably, the abundance of some identified toxins or virulence factors differs largely between the two groups of ST398 strains. In particular, the LA-ST398 strains produced on average higher amounts of Phospholipase C (Hlb) and the von Willebrand binding protein (vWbp). In contrast, the human-originated ST398 strains produced on average higher amounts of the CHIPS and SCIN proteins. In addition, a higher number of identified extracellular proteins (n = 114) from the LA-ST398 strains was involved in metabolic functions as compared to the human-originated ST398 isolates (n = 84). Collectively, these differences most likely reflect the different requirements for competitive success in the different ecological niches occupied by the LA-ST398 and the human-originated ST398 strains investigated in this study.

### Distinctive virulence factor signatures of LA-ST398 and human-originated ST398 strains

**Figure 4.**
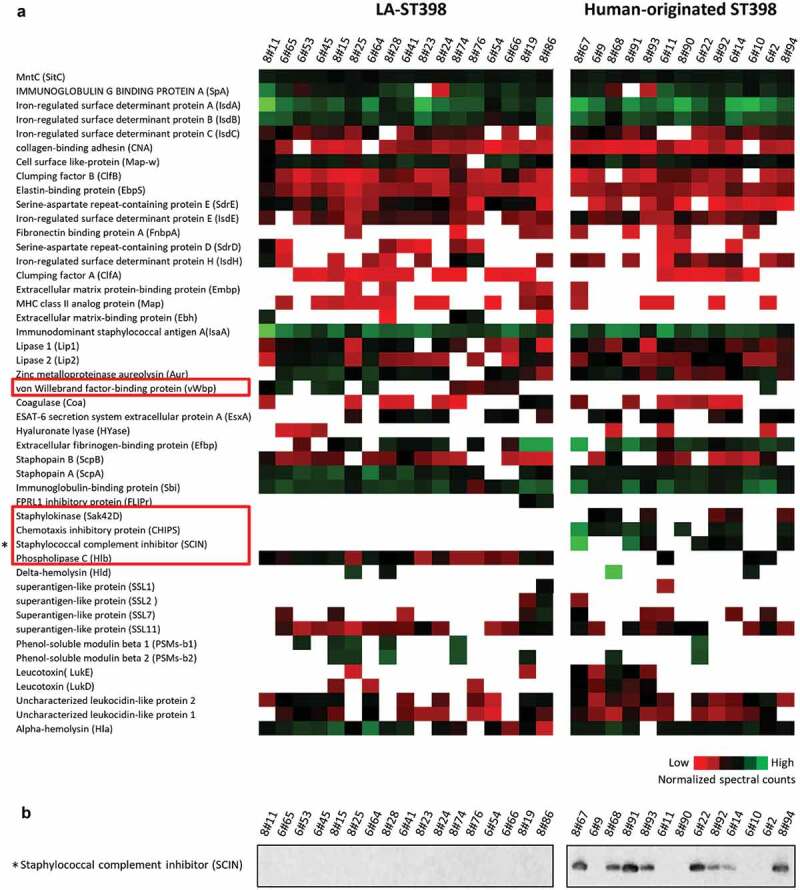
Extracellular virulence factors of the investigated LA-ST398 and human-originated ST398 strains. (a) A total number of 48 virulence factors was identified in all the investigated *S. aureus* ST398 isolates. Color-coded bars represent the identified proteins and their relative abundance based on normalized spectral counts. (b) The presence of the SCIN protein was assessed by Western blotting using the SCIN-specific monoclonal antibody 6D4.

To obtain more comprehensive insights into the pathogenic traits of the two groups of ST398 strains, the presence and levels of known virulence factors identified in the exoproteomes of the different investigated strains were inspected in detail. The relative abundance of these known virulence factors per exoproteome is presented in Figure 4. Overall, 47 distinct virulence factors were identified ranging from bacterial adhesion factors, exoenzymes, and immune evasion factors to toxins. Twenty of these factors are expressed to similar levels by at least 80% of the investigated strains, including Aur, ClfB, EbpS, IsdA, IsdB, MntC and SpA, with MntC being expressed at close to identical levels by all investigated isolates (Figure 4, top row). The remaining 27 virulence factors displayed a highly heterogeneous expression pattern among the investigated strains and this concerned, in particular, cytolytic toxins, such as Hlb, Hld, LukD, LukE, PSMβ1 and PSMβ2. Further, it is noteworthy that the three *pvl*-positive strains of clade 1.6b did not detectably produce the Panton-Valentine leukocidin (PVL)-toxin, which specifically affects human neutrophils and is commonly present among community-acquired MRSA strains. Likewise, the only strain carrying the enterotoxin gene *sea*, also belonging to clade 1.6b, did not detectably express this superantigen that has been implicated in food poisoning. On the other hand, several toxins (SSL1, SSL2, SSL7 and SSL11) belonging to the staphylococcal superantigen-like (SSL) family, which actually exhibit no super-antigen activity [41], were detected among the virulence factors produced by the investigated ST398 strains (Figure 4).

Most of the human-originated ST398 isolates were previously shown to harbor the β-hemolysin-converting prophage ϕSa3, which may carry the human-specific immune evasion cluster genes *chp, scn* and *sak* [6]. Indeed, the CHIPS protein was only detected in 9 of the 13 (70%) investigated human-originated ST398 strains. Further, the SCIN protein was identified in 7 (54%) of the human-originated strains and SAK was identified in 6 (46%) of the human-originated ST398 strains. Together, these observations imply that the CHIPS and SCIN proteins may be regarded as proteomic markers for the human-originated *S. aureus* ST398 population. This idea was verified by Western blotting with the SCIN-specific monoclonal antibody 6D4, using the same protein samples that were used for the MS analyses [27]. As shown in Figure 4(b), the SCIN protein was only detectable in human-originated isolates. In fact, the Western analysis detected SCIN also among the extracellular proteins of the human-originated strain 8#68, where this protein remained undetected by MS. However, in this respect one should bear in mind that a lack of identification of a particular protein by MS does not necessarily mean that this protein is completely absent from the respective sample. In contrast to the IEC proteins, the phospholipase C encoded by the *hlb* gene was identified in all of the LA-ST398 strains, whereas it was detected only in 2 (15%) of the human-originated ST398 strains. This implies that the phospholipase C may be regarded as a marker of the LA-ST398 population. A virulence factor that was predominantly detected in the LA-ST398 strains is the von Willebrand factor binding protein, which was identified in 10 (59%) of the LA-ST398 strains, whereas it was detected in only one of the investigated human-originated ST398 strains. Remarkably, the gene for this protein is present in all LA- and human-originated ST398 strains, so its detectable expression under the present experimental conditions is a more frequently occurring feature of the investigated LA-ST398 strains.

### A *Galleria mellonella* infection model reveals heterogeneity in the virulence of LA-ST398 and human-originated ST398 strains

**Figure 5.**
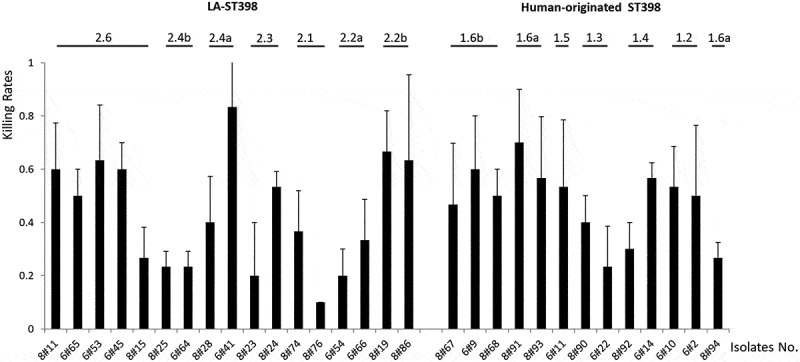
Virulence profile of the 30 investigated *S. aureus* ST398 isolates in *G. mellonella*. To profile the virulence of the investigated *S. aureus* ST398 strains, three independent *G. mellonella* infection experiments were performed. Per experiment, each investigated *S. aureus* ST398 strain was used to inoculate 10 *G. mellonella* larvae (30 larvae/strain in total). Each individual larva was inoculated with 2.5 × 10^5^ CFUs of the respective *S. aureus* ST398 strain. Larval killing was assessed at 48 h post inoculation. All values are the mean ± the standard deviation of the three independent infection experiments.

Since substantial exoproteome differences were observed between the LA-ST398 and the human-originated ST398 strains, we asked the question to what extent this exoproteome heterogeneity is reflected in the virulence of strains belonging to either of these two groups. As a first approach to answer this question, a *Galleria mellonella* larval infection model was employed in which the bacteria are challenged primarily by the innate immune system of the larvae. To this end, bacteria were cultured in RPMI medium to an OD_600_ of 0.5, collected by centrifugation, and washed and resuspended in PBS. Next, 10 μL aliquots of each strain (2.5 × 10^5^ CFU) were used to inoculate 30 larvae. As shown in Figure 5, at 48 h post infection the different investigated strains displayed substantial heterogeneity in larval killing. While isolates belonging to some particular sub-clades showed comparable larval killing within the respective sub-clade (e.g. 2.4b, 2.2b, 1.6a/b, and 1.2), strains belonging to other sub-clades showed quite distinct larval killing rates. To investigate which proteins could be associated with the observed intra-sub-clade differences in larval killing, the MS data of the respective strains were subjected to pairwise comparisons. Interestingly, a limited number of the identified extracellular proteins were significantly correlated with the killing of *G. mellonella* larvae. In particular, comparison of extracellular proteins from the more virulent LA-ST398 clade 2.2b with those from the less virulent clades 2.2a, 2.4b, 2.1 and 2.3 highlighted elevated extracellular levels of the immunoglobulin-binding protein Sbi, the MHC class II analog protein Map, coagulase and the N-acetylmuramoyl-L-alanine amidase Sle1 for strains of clade 2.2b (Supplementary Table S3). Also, the strains of clade 2.2b were the only ones to detectably produce the FPRL1 formyl peptide receptor-like 1 inhibitor FLIPr (Figure 4(a)). Furthermore, comparison of extracellular proteins from the more virulent LA-ST398 clade 2.6 with those from the less virulent clades 2.2a, 2.4b, 2.1 and 2.3 highlighted elevated extracellular levels of Sbi, Sle1, and aureolysin for strains belonging to clade 2.6 (Supplementary Table S3). In contrast, comparisons of human-originated ST398 strains with different virulence resulted only in the detection of significantly elevated extracellular aureolysin levels for strains belonging to clade 1.2.

**Figure 6.**
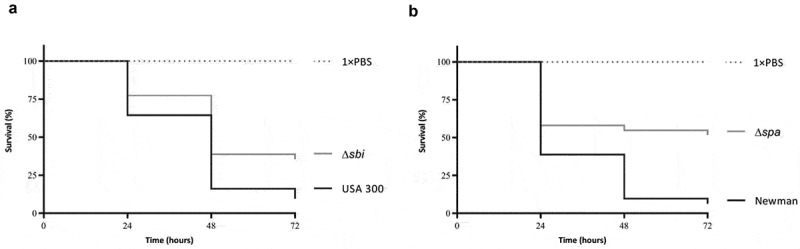
Attenuation of Sbi- and SpA-deficient *S. aureus* strains in the *G. mellonella* infection model. (a) Survival curves of *G. mellonella* larvae (n = 30) inoculated with 2.5 × 10^6^ CFUs of *S. aureus* strain USA 300 or the isogenic *sbi* mutant strain. (b) Survival curves of *G. mellonella* larvae (n = 30) inoculated with 2.5 × 10^6^ CFUs of *S. aureus* strain Newman or the isogenic *spa* mutant strain. Larval survival was assessed at 24, 48 and 72 h post infection. The statistical significance of the observed differences in the larval survival was assessed using a Wilcoxon test (Δ*sbi* versus USA 300, P = 0.0484; Δ*spa* versus Newman, P = 0.0068).

To verify possible effects of some of these extracellular proteins on virulence, we tested *sbi* and *spa* single mutant strains along with the respective parental strains in the *G. mellonella* infection model. Of note, since the respective *S. aureus* ST398 strains were not available, we applied an *sbi* mutant of *S. aureus* USA300 and a *spa* mutant of *S. aureus* Newman for these analyses. Specifically, each of the investigated strains was used to infect 30 larvae, where a bacterial suspension of 10 μL containing 2.5 × 10^6^ CFUs in PBS was used per larval inoculation. Subsequently, the larval mortality was monitored over 72 h. As shown in Figure 6, both the *sbi* and *spa* mutants displayed significant lower killing rates compared to the respective wild-type. This implies that both the SpA and Sbi proteins contribute to larval killing activity, as was predicted based on the correlation of the present exoproteome data to the killing of *G. mellonella* larvae.

### Human-originated ST398 strains are on average more cytotoxic than LA-ST398 strains in a HeLa cell infection model

**Figure 7.**
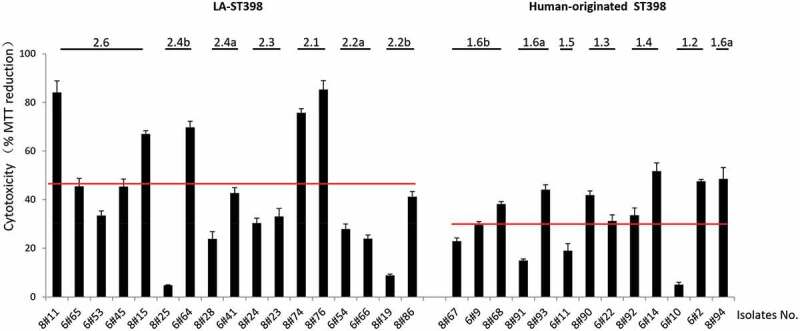
Cytotoxicity profile of the 30 investigated *S. aureus* ST398 isolates in HeLa cells. Hela cells were infected with bacteria at a MOI of 50:1. Upon 3 h incubation, the non-internalized bacteria were eliminated by washing the cells twice in fresh medium, and by bacterial killing with lysostaphin immediately after the last washing step. Subsequently, the HeLa cell viability was assayed by measuring the reduction of MTT. The results are presented as the percentage of MTT reduction relative to the uninfected control. The cytotoxicity of each *S. aureus* ST398 strain was assessed in three independent experiments. The two red lines mark the average MTT reduction upon HeLa cell infection with the investigated LA-ST398 or human-originated ST398 strains, respectively.

To gain insights into the cytotoxicity of LA-ST398 strains and human-originated ST398 strains in a nonprofessional phagocyte infection model, we employed HeLa cells. These cells were challenged at a MOI of 50 with PBS-diluted *S. aureus* that had been cultured in RPMI medium as described above for the *G. mellonella* infection experiments. Upon 3 h incubation, any non-internalized bacteria were eliminated by washing the cells twice in fresh medium, and by bacterial killing with lysostaphin immediately after the last washing step. Subsequently, the cell viability was assayed by measuring the reduction of MTT. As shown in Figure 7, the LA-ST398 strains displayed on average a higher cytotoxicity than the human-originated ST398 strains. Further, in both groups of strains we observed heterogeneity in cytotoxicity, but a greater extent of heterogeneity was observed for the LA-ST398 strains. Intriguingly, the observed variations in cytotoxicity cannot be directly reconciled with the patterns of virulence factors as presented in Figure 4(a). This most likely means that the observed cytotoxicity relates to the combined effects of multiple virulence factors, which obscures the impact of individual virulence factors.

Notably, contrary to the results obtained in the *G. mellonella* infection model, isolates belonging to most investigated sub-clades showed comparable levels of cytotoxicity within the respective sub-clade (e.g. 2.4a, 2.3, 2.1, 2.2a, 1.6b, 1.3 and 1.4). Only strains belonging to the sub-clades 2.6, 2.4b, 2.2b and 1.2 displayed highly distinct cytotoxicity in the HeLa cell infection model. To investigate which proteins could be associated with the observed intra-sub-clade differences in cytotoxicity, the MS data of the respective strains were first subjected to pairwise comparisons. In particular, a comparison of the extracellular proteins from the more cytotoxic LA-ST398 clade 2.3 and 2.2b with those from the less virulent clade 2.1 highlighted elevated extracellular levels of the proteins Map, Sbi and coagulase (Supplementary Table S3). However, other comparisons did not pinpoint particular virulence factors as being critical for cytotoxicity of the LA-ST398 or human-originated ST398 strains.

### HeLa cell killing by purified SCIN and CHIPS proteins

**Figure 8.**
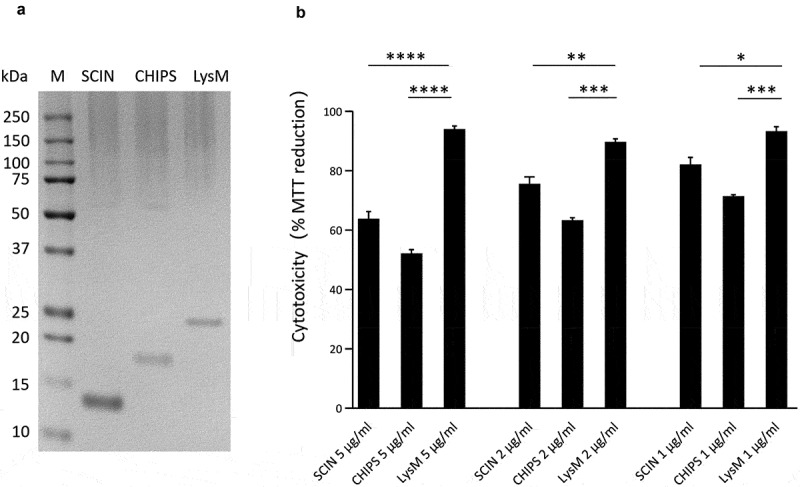
Cytotoxicity of the SCIN and CHIPS proteins. (a) LDS-PAGE analysis of the purified *S. aureus* SCIN and CHIPS proteins, and the LysM domain of the *S. aureus* Sle1 protein. (b) To assay the cytotoxicity of SCIN, CHIPS and LysM domain, HeLa cells were incubated with different amounts of the purified proteins for 24 h. Subsequently, the viability of the HeLa cells was assayed by measuring the reduction of MTT. The statistical significance of the observed differences in the killing of HeLa cells was assessed using one-way ANOVA and a subsequent Dunnett correction to adjust the P-values (SCIN 5 μg/ml vs. LysM 5 μg/ml, P < 0.0001; CHIPS 5 μg/ml vs. LysM 5 μg/ml, P < 0.0001; SCIN 2 μg/ml vs. LysM 2 μg/ml, P = 0.0053; CHIPS 2 μg/ml vs. LysM 2 μg/ml, P = 0.0003; SCIN 1 μg/ml vs. LysM 1 μg/ml, P = 0.0102; CHIPS 1 μg/ml vs. LysM 1 μg/ml, P = 0.0006). *, P < 0.05; **, P < 0.01; ***, P < 0.001; ****, P < 0.0001; P < 0.05 was considered significant.

As is clearly evident from Figure 4, a common feature of many human-originated ST398 isolates is the production of the IEC proteins SCIN and CHIPS. Therefore, we investigated to what extent these two virulence factors may contribute to the staphylococcal cytotoxicity in our HeLa cell infection model. To this end, the SCIN and CHIPS proteins were heterologously overexpressed with a His_6_-tag in *L. lactis* and purified by metal affinity chromatography as shown in Figure 8(a). As a control, the LysM subdomain of the *S. aureus* Sle1 protein was similarly overproduced in *L. lactis* and purified (Figure 8(a)). Of note, it was anticipated that this LysM domain would not be cytotoxic based on previous studies where possible medical applilcations of LysM domains were investigated [42]. Subsequently, HeLa cells were incubated for 24 h with different amounts of the purified proteins and MTT reduction was subsequently assayed to evaluate the HeLa cell viability. As shown in Figure 8(b), the incubation of HeLa cells with either SCIN or CHIPS resulted in a significant reduction of HeLa cell viability compared to the negative control protein LysM, and this effect was concentration-dependent. This observation shows that both SCIN and CHIPS are cytotoxic for HeLa cells, and it implies that these two proteins contribute to some extent to the higher cytotoxicity of the investigated human-originated ST398 strains.

## Discussion

The successful adaptation of *S. aureus* ST398 to different host niches in combination with the acquisition of genes for a wide range of different virulence factors has turned this staphylococcal lineage into a serious threat for public health. This is underscored by epidemiological and genetic studies, which uncovered considerable variations in staphylococcal ST398 isolates, especially with respect to MGEs, such as prophages, plasmids and pathogenicity islands [6,8,11]. Nevertheless, our understanding of the actual expression of virulence factors by strains belonging to the ST398 lineage and their collective impact on overall pathogenicity is still limited. Therefore, in the present study, we performed a large-scale exoproteome comparison for 30 clinical *S. aureus* ST398 strains in combination with an assessment of their virulence. These 30 strains, which were isolated in Europe and China represent a wide range of epidemiological and genomic backgrounds. An important outcome of the present study is the identification of proteomic signatures for staphylococcal virulence and host adaptation. In addition, the comparative analyses guided the identification of the Sbi, SpA, SCIN and CHIPS proteins as important staphylococcal virulence factors in different infection scenarios.

While the 30 investigated strains show a high degree of genomic relatedness, our MS analyses still revealed substantial heterogeneity in their exoproteomes. In fact, merely 40 extracellular proteins were found to be produced by all investigated ST398 isolates, whereas 131 proteins were identified in only one or two of these isolates. This is reminiscent of what was previously observed for a set of genetically tightly related isolates of *S. aureus* with the *spa* type t437 from Europe and China [40]. However, the *S. aureus* isolates with *spa* type t437 showed also substantial variation in the overall numbers of identified extracellular proteins, whereas this variation was not observed for the here investigated *S. aureus* ST398 isolates. On the contrary, rather homogeneous exoproteome patterns were previously observed for isolates of the USA300 lineage from the Copenhagen area in Denmark. Nonetheless, the variations that were observed in the exoproteomes of these USA300 isolates distinguished HA-MRSA isolates from CA-MRSA isolates [43]. Together, the previous and present observations suggest that the degree of exoproteome heterogeneity displayed by strains belonging to particular *S. aureus* lineages depends, most likely, on the respective lineage, their geographical distribution, and the host from which they were isolated.

Notably, the observed exoproteome heterogeneity in strains belonging to *S. aureus* ST398 or *spa* type t437 was mostly related to a differential abundance in the identified ECPs. The release of typical cytoplasmic proteins into the culture supernatant is a common physiological feature of clinical isolates of *S. aureus* and many other microorganisms [24,37,44]. Among the 495 extracellular proteins that we identified for *S. aureus* ST398, ~63% were predicted to be located in the cytoplasm, and these ECPs represented ~48% of the extracellular protein abundance. Of note, the numbers of identified ECPs differed substantially among the 30 investigated ST398 isolates, ranging from 51 to 246. In contrast, a recent exoproteome comparison for 18 *S. aureus* isolates belonging to CC8, CC22 and CC398 revealed a rather homogeneous protein pattern with 607 identified cytoplasmic proteins accounting for ~70% of the identified proteins, but only for ~13% of the extracellular protein abundance [38]. Such variations may reflect the existence of different mechanisms in the excretion of ECPs, which include destabilization of the cell envelope caused by autolysins like Atl, prophage activity and/or cytolytic toxins [45]. In the present data set, variations in the numbers of different identified ECPs cannot be correlated to Atl because the exoproteomes of the different investigated strains contained comparable amounts of Atl (Supplementary Table S1). This implies that Atl is not a major contributor to the excretion of ECPs by ST398 strains. On the other hand, three phage-associated proteins, namely the major tail protein (B9DJ00), the major capsid protein (M1SVC5) and the phage infection protein (Q5HKT0), were exclusively detected in those isolates that displayed the highest abundance of ECPs (Supplementary Table S1). This observation is fully in line with our previous observation that the detection of ECPs of *S. aureus* strains with *spa* t437 was correlated with phage activity. Clearly, this is not the case for all *S. aureus* lineages, as Pasztor *et al*. demonstrated that the elimination of prophages ϕ11, 12 and 13 from the *S. aureus* strain 8325–4 had no marked influence on the release of ECPs [45]. Another mechanism that facilitates the appearance of ECPs concerns the expression of α-type phenol-soluble modulins (PSMα), which weaken the cytoplasmic membrane of the producing bacteria, resulting in the release of not only ECPs, but also lipids, nucleic acids and ATP [46]. At present, we cannot exclude the possibility that the PSMα toxins also contribute to the release of ECPs by ST398 strains, because these very small proteins (20–30 residues) are difficult to detect by MS. Lastly, it is important to note that it was recently shown for isolates of the USA300 lineage that specific patterns of ECPs reflect metabolic niche adaptations that are governed by differential activity of major staphylococcal regulators of gene expression [47]. It is well conceivable that differential gene regulation is, at least in part, also responsible for the here observed exoproteome heterogeneity.

Presumably, the *S. aureus* ST398 lineage has gone through at least two host changes in its evolutionary history, being transmitted from humans to livestock and, more recently, back to the human host [7]. The accompanying adaptations were driven by the specific conditions in the “new” host and the challenges imposed by the new host’s immune defenses. This imposed the need for altered expression of virulence factors and/or the acquisition of new virulence factors necessary for successful adaptation to the new host. This appears to be reflected by the combined genomic and proteomic data. For instance, the phylogeny of the investigated ST398 strains based on the core genome as presented in Table 1 suggests that the human-originated strains are genetically more diverse than the LA-ST398 strains, whereas the exoproteomes of the human-originated strains are less diverse.

A major aim of our current study was to pinpoint critical changes in the exoproteome that reflect specific host adaptations to the animal or human host settings. Thus, we made a first attempt to correlate our exoproteome data to the bacterial virulence as determined with the help of two different infection models, namely the larvae of the wax moth *G. mellonella* and the human HeLa cell line. Infection outcomes observed in the *G. mellonella* infection model were previously shown to correlate well with the outcomes in murine infection models for a number of different opportunistic human pathogens [20]. However, in contrast to mice, *G. mellonella* is only capable of mounting innate immune responses with the aid of phagocytic cells [48]. Consistent with this view, it was intriguing to see that the correlation of quantitative proteomic signatures to virulence in the *G. mellonella* infection model highlighted the Sbi protein as an important virulence factor, a cue that was subsequently confirmed by infecting larvae with a *sbi* mutant strain that was shown to be attenuated in larval killing. Sbi is an immunoglobulin-binding protein produced by many strains of *S. aureus*, which was previously characterized as an immune evasion factor that helps bacteria to avoid innate immune defenses via interfering with opsonophagocytosis [49]. Possibly, this is also the case when *S. aureus* infects *G. mellonella*, although indirect effects due to the absence of Sbi on the surface of infecting bacteria cannot be excluded. Similar to Sbi, also the SpA protein was associated with larval killing in the *G. mellonella* model and a role for SpA in the infective process was subsequently verified by showing that *S. aureus* cells lacking the *spa* gene were attenuated. In fact, this result is consistent with previous studies showing that SpA is an important determinant for *S. aureus* virulence in a murine septic arthritis model [50]. It is noteworthy that the experiments showing the roles of Sbi and Spa in larval killing were performed with strains that belong to other lineages than *S. aureus* ST398 (i.e. USA300 and Newman) and that, nonetheless, the attenuation phenotype predicted based on our proteome analyses with *S. aureus* ST398 strains was observed. This suggests that our present observations also have broader implications for other lineages of *S. aureus*. For the HeLa cell model, it turned out somewhat harder to link particular extracellular virulence factors to cytotoxicity, but it is noteworthy that similar to the *G. mellonella* model the Sbi protein was recognized as one of the distinguishing features for HeLa cell cytotoxicity. Importantly, the HeLa cell infection model also allowed validation of the involvement in cytotoxicity of the *S. aureus* CHIPS and SCIN proteins, which are exclusively produced by human-originated ST398 strains. Consistent with the view that the human-originated ST398 strains are overall more cytotoxic than the LA-ST398 strains, it was observed that the purified CHIPS and SCIN proteins are toxic for HeLa cells, unlike the purified control protein. While CHIPS and SCIN are very well-characterized as immune evasion proteins [12], the observation that they may have cytotoxic properties is novel. Interestingly, the here detected cytotoxicity of CHIPS and SCIN is fully in line with results from a recent study where the prophage ϕSa3, which carries the *chp* and *scn* genes, was implicated in *S. aureus* ST398 virulence in a *Caenorhabditis elegans* infection model [51]. Altogether, these findings highlight the critical roles of the SCIN and CHIPS proteins in staphylococcal virulence.

## Conclusion

The present study highlights the proteomic signatures that distinguish strains belonging to the livestock-associated and human-originated *S. aureus* ST398 lineage. A remarkable observation was that the exoproteomes of the human-originated strains were more similar to each other compared to the exoproteomes of the LA-ST398 strains, despite the fact that the latter strains were more closely related to each other. This is suggestive of particular proteomic adaptations being crucial for the reintroduction of *S. aureus* ST398 from livestock into the human population. Among these adaptations, but apparently not strictly required, is the acquisition of the immune evasion proteins CHIPS, SCIN and Sak. Of note, our present study shows that at least CHIPS and SCIN do have some cytotoxic properties next to their established roles in immune evasion by, respectively, inhibiting chemotaxis and complement. Further, the here presented experiments with the *G. mellonella* infection model uncovered a, thus far, overlooked involvement of the immune evasion proteins Sbi and SpA in the staphylococcal killing of larvae. Whether this reflects direct or indirect effects of Sbi and SpA remains to be shown, but the observed effects are fully consistent with the established roles of these proteins in staphylococcal infection in humans.

## Supplementary Material

Supplemental Material

Supplemental Material

Supplemental Material
